# Bioabsorption and effectiveness of long-lasting permethrin-treated uniforms over three months among North Carolina outdoor workers

**DOI:** 10.1186/s13071-019-3314-1

**Published:** 2019-01-23

**Authors:** Kristin M. Sullivan, Alison Poffley, Sheana Funkhouser, Jeffrey Driver, John Ross, Maria Ospina, Antonia M. Calafat, Charles B. Beard, Avian White, Jo Anne Balanay, Stephanie Richards, Megan Dyer, Thomas N. Mather, Steven Meshnick

**Affiliations:** 1 Department of Epidemiology, University of North Carolina Gillings School of Global Public Health, Chapel Hill, NC USA; 2 Risksciences LLC, Longboat Key, FL and University of South Florida, College of Public Health, Tampa, FL USA; 3 Division of Laboratory Sciences, National Center for Environmental Health, Centers for Disease Control and Prevention, Atlanta, GA USA; 4 Division of Vector-borne Diseases, Centers for Disease Control and Prevention, Ft. Collins, CO USA; 5 Environmental Health Sciences Program, Department of Health Education and Promotion, East Carolina University, Greenville, NC USA; 6 Center for Vector-Borne Disease, University of Rhode Island, Kingston, RI USA

**Keywords:** Permethrin, Permethrin treatment, Permethrin absorption, Repellent, Ticks, Tick bites, Mosquitoes

## Abstract

**Background:**

Vector-borne diseases are an important cause of morbidity and mortality in the USA. Effective, convenient prevention methods are needed. Long-lasting permethrin-impregnated (LLPI) clothing can prevent tick bites, however, additional information is needed on the real-world effectiveness and safety of this preventative measure.

**Methods:**

In this pilot study, we recruited state and county park employees from North Carolina to wear LLPI uniforms for three months during the summer of 2016. We collected spot urine samples for biomonitoring of permethrin metabolites at one week, one month and three months after first use of the LLPI uniform. Following three months of wear, we collected pants and socks and analyzed them for permethrin content and mortality to ticks and mosquitoes.

**Results:**

Thirteen park employees were included in the analysis. Bioactive amounts of permethrin remained in all clothing swatches tested, although there was great variability. Tick mortality was high, with 78% of pant and 88% of sock swatches having mean knockdown percentages ≥ 85%. In contrast, mosquito mortality was low. Over the study period, the absorbed dosage of permethrin averaged < 4 μg/kg/d of body weight based on measurements of three metabolites.

**Conclusions:**

LLPI clothing retained permethrin and bioactivity against ticks after three months of use in real-world conditions. The estimated absorbed dosage of permethrin was well below the U.S. EPA level of concern, suggesting that LLPI clothing can be used safely by outdoor workers for tick bite prevention.

**Electronic supplementary material:**

The online version of this article (10.1186/s13071-019-3314-1) contains supplementary material, which is available to authorized users.

## Background

Vector-borne diseases are an important cause of morbidity and mortality in the USA. Tick-borne diseases predominate, accounting for over 75% of all vector-borne diseases reported to the Centers for Disease Control and Prevention in recent years [1]. During 2016, 48,610 cases of tickborne disease, including 36,429 Lyme disease cases, were reported [1]. Recent trends indicate that the burden of both tick-borne and mosquito-borne diseases may be growing. The number of reported cases of tick-borne disease, the geographical range of ticks, and the number of tick-borne pathogens have increased in recent years [1–3]. In addition, the recent emergence of Zika and chikungunya viruses in the Americas highlights the increasing threat of mosquito-borne diseases within the USA [4–6]. To combat this mounting public health threat, tick and mosquito bite prevention measures that are both effective and easy to use are needed.

One method of protection is the use of the insecticide permethrin on clothing which may come in contact with ticks and mosquitoes. Permethrin can be self-applied to clothing using aerosol spray. This application method is effective but sub-optimal because it requires frequent reapplication and adherence is often poor [7, 8].

An alternative to self-application of permethrin is factory-impregnation. Factory-based, long-lasting permethrin-impregnated (LLPI) clothing is EPA-registered [9] and has been shown to maintain repellency for up to 70 washes [10]. For this reason, LLPI clothing may be preferable among people with frequent, long-term potential exposure to ticks and mosquitoes. In a randomized control study among outdoor workers, the use of LLPI clothing was shown to be 82% effective in reducing tick bites for up to one year [11]. However, the duration of protection by LLPI clothing worn in real-world conditions is unknown.

LLPI clothing yields a reasonable certainty of no harm to wearers [9, 12], as permethrin is poorly absorbed through the skin when applied directly in solvent [13] and is even less bioavailable when sorbed to clothing [9, 14]. However, there have been few long-term safety studies among LLPI clothing wearers.

In this study, we aimed to (i) evaluate the permethrin content that remained in LLPI clothing after three months of routine use; (ii) determine the lethality of worn clothing to ticks and mosquitoes after three months of use; and (iii) determine the average absorbed dosage of permethrin among LLPI clothing wearers over a three-month period.

## Methods

### Participants

Thirteen participants were recruited between May and July 2016 from outdoor workers at the North Carolina Divisions of Parks & Recreation and Wake County Parks. Any worker was eligible to enroll in the study if s/he was over the age of 18, wore a uniform for at least four days each week during the spring/summer season and gave informed consent. Workers were excluded if they were pregnant or planned to become pregnant in the next year, had a known allergy or sensitivity to insecticides or were non-English speakers. Non-English speakers were excluded from this pilot study because study staff only spoke English and would not have been able to obtain consent. Study personnel contacted workers and invited them to participate in the study.

### Study procedures

At baseline, participants were asked to (i) complete a questionnaire regarding demographic information and insect repellent use; and (ii) send spring or summer uniform items (except underwear/briefs) to Insect Shield Inc. (Greensboro, North Carolina) for standard LLPI treatment. Participants sent half of their uniforms to be treated and, upon receiving those back, sent the other half. Participants were instructed to notify study personnel if they ordered new clothing during the study.

Spot urine samples were collected from each of the participants at four points during the study: before wearing LLPI clothing; after one week of first LLPI clothing use; after one month of first LLPI clothing use, and after three months of first LLPI clothing use. Urine samples were aliquoted and stored at -80 °C and then shipped to the CDC environmental health laboratory on dry ice.

After three months of LLPI clothing use, participants completed a follow-up questionnaire indicating frequency of uniform wear, hours worked outdoors, uniform laundering practice, use of insecticides or repellents, and tobacco use. At this time, participants were also asked to submit one set of uniform items to study personnel. Collected items were first sent to the University of Rhode Island for tick killing efficacy studies and then sent to East Carolina University for mosquito knockdown/mortality and permethrin retention studies.

### Laboratory analysis of worn LLPI clothing

Participant-worn clothing, along with control clothing, was evaluated for permethrin retention and tick and mosquito knockdown/mortality. Three swatches from each pair of submitted pants and two swatches from each pair of submitted socks were collected. For treated controls, a single swatch was collected from each of three pairs of permethrin-treated but unworn control pants and each of two permethrin-treated but unworn control socks. For untreated controls, a single swatch was collected from each of three pairs of untreated, unworn control pants and each of two untreated, unworn control socks.

#### Tick mortality

To determine tick mortality, three swatches of clothing were placed in the horizontal plane on a benchtop (tick-exposure side up) as described in Eisen et al. [15]. Ten laboratory-reared, pathogen-free *Ixodes scapularis* nymphs were placed on the fabric for three min. The nymphs were then collected and held in plastic vials which were placed into a humidity chamber (> 85% RH; 21°C) and checked at 24 h for survival. After blowing on the ticks to stimulate movement, a visual test of survival was performed and the number of nymphs that showed signs of life, mainly through movement, was recorded. This procedure was repeated for all swatches of pants and socks.

#### Mosquito knockdown and mortality

*Aedes aegypti* (generation F_3_ originating from Florida), classified as resistant to permethrin, and *Aedes albopictus* (generation F_10_ originating from Georgia), classified as susceptible to permethrin, were used to test mosquito knockdown (2 h post-exposure to fabrics) and mortality (24 h post-exposure to fabrics). The mosquito bioassays were conducted using a Petri dish exposure method as described in Richards et al*.* [16]. Briefly, mosquitoes were exposed to treated fabric swatches (8.5 cm diameter) cut from clothing that had been worn by outdoor workers. Mosquitoes were also exposed to unworn treated and unworn control fabric swatches. Swatches were placed into individual petri dishes and mosquitoes (*n* = 3–8/dish) were immobilized with cold in a -20 °C freezer for *c.*45 s before being transferred to each dish. In a few cases, some mosquitoes were harmed (human error) during the transfer and hence were excluded from analyses. After mosquitoes were exposed to swatches for two min, the Petri dish was chilled and mosquitoes were transferred into 0.25 l cardboard cages (separated by treatment) with mesh screening. Mosquitoes were then provided a 20% sucrose solution and incubated with 80% humidity at 28 °C and 14:10 h light:dark cycle. Knockdown (i.e. lying down or unable to fly) was assessed for each control and treatment group at 2 h post-exposure, and mortality was assessed 24 h post-exposure to fabric swatches.

#### Permethrin retention

The swatches used for mosquito studies were used to test the amount of permethrin retention. Permethrin content was determined as described in Richards et al. [16]. Briefly, fabric swatches were transferred to separate amber glass vials containing 40 ml acetone and soaked for one h to extract permethrin in a water-filled Sonicator (Ultrasonic Bath, Fisher Scientific, Kennesaw, GA). Extracts (1.5 ml) from swatches were transferred to 1.5 ml amber GC vials and 1 μl of the extract was analyzed directly by capillary gas chromatography with flame ionization detector (GC-FID) using an Agilent GC 6850 (Agilent Technologies, Alpharette, GA).

### Laboratory analysis of urine samples

Urine samples were analyzed for three permethrin metabolites, specifically cis-3-(2,2-dichlorovinyl)-2,2-dimethylcyclopropane carboxylic acid (cis-DCCA), trans-3-(2,2-dichlorovinyl)-2,2-dimethylcyclopropane carboxylic acid (trans-DCCA) and 3-phenoxybenzoic acid (3-PBA). These target analytes were extracted and concentrated from urine by off line solid phase extraction, separated by high-performance liquid chromatography using a gradient elution program and analyzed by isotope dilution tandem mass spectrometry as described previously [17]. Accuracy and precision for each analytical run were monitored through the use of calibration standards, reagent blanks, and quality control materials. Limits of detection for cis-DCCA, trans-DCCA, and 3-PBA were 0.5, 0.6 and 0.1 μg/l, respectively. Urinary creatinine (mg/dl) was also measured at CDC using an enzymatic reaction. We replaced metabolite concentrations < LOD with a value equal to the LOD for the statistical analysis.

### Calculation of absorbed dose

The measured metabolite concentrations were used to estimate the absorbed dose of permethrin. The stoichiometry of metabolites from permethrin was obtained from Ratelle et al. [18] based on a human metabolism study. The absorbed dosage was estimated from metabolite concentrations as:

1 $$ \frac{\mathrm{Dosage}={\mathrm{Metabolite}}^{\ast}\left(\mathrm{Urine}\ \mathrm{excreted}\right)\ast \left({\mathrm{MW}}_{\mathrm{permethrin}}/{\mathrm{MW}}_{\mathrm{metabolite}}\right)}{\left(\mathrm{Fraction}\ \mathrm{in}\ {\mathrm{urine}}^{\ast}\mathrm{Bodyweight}\right)} $$

where Dosage = micrograms permethrin absorbed per kilogram bodyweight; Metabolite = micrograms of metabolite per liter of urine (see Table 1); Urine excreted = liters of urine excreted per day (assumed to be 1.5 l/day as in [12]); MW_permethrin_ = the molecular weight of permethrin (391 daltons); MW_metabolite_ = the molecular weight of metabolite (see Table 1); Fraction in urine = the fraction of dose excreted in urine as metabolite (see Table 1); and Bodyweight = bodyweight in kilograms (assumed to be 70 kg).

**Table 1:** Summary of metabolite urine concentration and three-month average estimated permethrin quantities from 12 participants^a^

		Median metabolite urine concentrations (μg/l)				3-month average estimated permethrin (from Eqn 1)			
Metabolite	Metabolite molecular weight	Baseline	1 week	1 month	3 months	3-month average^a^	Dose fraction in urine^b^	Urine concentration (μg/l)	Dosage (μg/kg)
3-PBA	214	1.6	46.7	47.1	15.3	36.4	0.46	36.4	3.1
Cis-DCCA	209	< 0.5	22.1	29.7	10.2	20.7	0.43	20.7	3.2
Trans-DCCA	209	< 0.6	57.4	58.8	21.6	45.9	0.26	45.9	4.3

^a^Mean of 1 week, 1 month and 3-month average measurements, across all participants; human excretion of metabolites following a known oral dose were measured by Ratelle et al. [18]

^b^Human excretion of metabolites following a known oral dose were measured as in Ratelle et al. [18]

### Statistical analyses

The pharmacokinetics of excretion of permethrin metabolites was analyzed as described by Ratelle et al. [18] to calculate the absorbed permethrin dosage. We analyzed mosquito mortality using Pearson’s chi-square tests to evaluate independence in contingency table analyses and used an alpha level of 0.05 for significance testing. Analysis of variance (ANOVA) was used to evaluate differences in permethrin content between fabric swatches. Permethrin quantities were log-transformed (x + 1) prior to using ANOVA to improve normality. We calculated the Pearson’s correlation coefficient to evaluate the linear relationship between pant and sock permethrin content. SAS 9.4 was used for general numerical and graphical analysis.

## Results

The thirteen participants were asked to submit urine at four time points during the study and submit worn LLPI clothing at study termination. Eight participants submitted both clothing and urine, four participants submitted only urine and one participant submitted only clothing before dropping out because of suspected permethrin allergy.

### Permethrin retention

Nine participants submitted a total of 17 articles of clothing for analysis: 9 pairs of pants and 8 pairs of socks. Untreated clothing and Insect Shield-treated but unworn clothing were used as controls. In all articles of worn clothing, bioactive amounts of permethrin remained after three months of use (Table 2).

**Table 2:** Summary of urine metabolite, permethrin retention and tick and mosquito knockdown/mortality results among 13 participants

Control/participant ID	Permethrin equivalent daily dose (μg/kg/day). Multi-month average^a^			Clothing type	Mean ± SD permethrin content in μg/cm^2b^	Mean tick mortality (%)^c^	Mean *Ae. albopictus* knockdown (2 h) and mortality (24 h) (%)^d^		Mean *Ae. aegypti* knockdown (2 h) and mortality (24 h) (%)^d^	
	3-PBA	cis-DCCA	trans-DCCA				2 h	24 h	2 h	24 h
Untreated controls	–	–	–	Pants	0.0 ± 0.0	3	0	0	0	0
				Socks	3.5 ± 0.2	7	0	0	0	0
Treated controls	–	–	–	Pants	14.2 ± 1.6	100	11	0	0	0
				Socks	48.5 ± 0.2	100	15	0	0	0
NC01	3.24	2.99	3.98	Pants	10.2 ± 1.5	100	12	0	0	0
				Socks	24.7 ± 13.8	97	0	0	0	0
NC02	1.72	1.14	2.66	Pants	4.9 ± 0.3	90	0	0	0	0
				Socks	13.9 ± 0.8	97	10	10	0	0
NC03	0.75	0.37	0.97	Pants	3.1 ± 0.4	17	7	7	0	8
				Socks	5.5 ± 1.2	97	0	0	0	0
NC04	1.88	0.67	2.34	–	–	–	–	–	–	–
				–	–	–	–	–	–	–
NC05	0.54	0.26	0.64	Pants	41.8 ± 2.3	100	100	20	13	13
				Socks	68.5 ± 5.6	100	0	0	0	0
NC06	7.12	4.64	9.66	Pants	27.6 ± 7.6	97	20	7	0	0
				Socks	24.8 ± 0.7	40	0	0	0	0
NC07	0.33	0.19	0.40	Pants	3.4 ± 0.3	47	0	0	7	0
				Socks	89.1 ± 42.4	100	25	0	0	0
NC08	0.86	0.39	1.03	–	–	–	–	–	–	–
				–	–	–	–	–	–	–
NC09	0.32	0.12	0.36	–	–	–	–	–	–	–
				–	–	–	–	–	–	–
NC10	2.10	1.14	2.54	–	–	–	–	–	–	–
				–	–	–	–	–	–	–
NC11	–	–	–	Pants	13.0 ± 1.5	87	0	0	0	0
				Socks	–	–	–	–	–	–
NC12	0.79	0.51	1.08	Pants	20.3 ± 1.1	100	33	0	0	0
				Socks	54.7 ± 15.8	97	0	0	0	0
NC13	1.29	1.40	2.08	Pants	20.2 ± 2.0	100	89	0	11	0
				Socks	53.7 ± 3.7	97	0	0	0	0

^a^Three urine metabolites were tested: 3-PBA: 3-Phenoxybenzoic acid; cis-DCCA: Cis-3-(2,2-dichlorovinyl)-2,2-dimethylcyclopropane carboxylic acid; and trans-DCCA: Trans-3-(2,2-dichlorovinyl)-2,2-dimethylcyclopropane carboxylic acid

^b^Mean permethrin content was calculated as the mean of either three pants swatches tested from the same pair of pants or two sock swatches tested (one from each sock in a pair)

^c^Mean tick mortality indicates the mean percentage of ticks assessed as dead from visual inspection at 24 h. The mean was determined from either the three pants swatches tested or the two sock swatches tested

^d^Mean mosquito knockdown/mortality indicates the mean percentage of mosquitoes assessed as knocked down at 2 h and dead at 24 h. The mean was determined from either the three pants swatches tested or the two sock swatches tested

Unworn, treated control pants and socks retained relatively high amounts of permethrin after three months, averaging 14.2 μg/cm^2^ in three pant swatches and 48.5 μg/cm^2^ in two sock swatches. Unworn, untreated swatches from pants and socks had low but non-zero, permethrin contents averaging 0.0 and 3.5 μg/cm^2^, respectively. All clothing had mean permethrin contents above the untreated controls. Over 40% of worn pant swatches and 50% of worn sock swatches retained permethrin above the level of the unworn treated control.

**Fig. 1:**
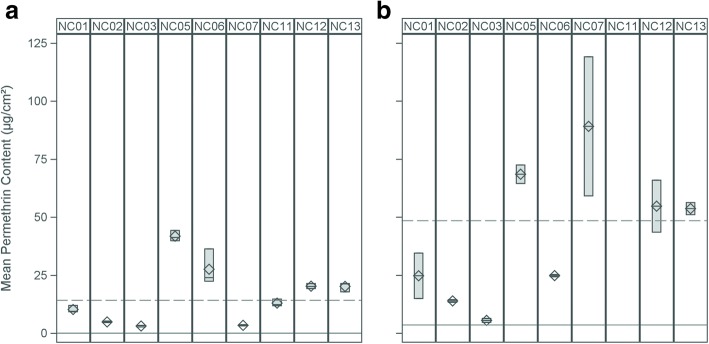
Permethrin content retained in LLPI pants and socks after three months of wear. **a** Range (colored box), mean (diamond) and median (bar) of permethrin content (μg/cm^2^) retained in three swatches from a single pair of pants submitted by nine park employees following three months of use. **b** Range (colored box), mean (diamond) and median (bar) of permethrin content (μg/cm^2^) retained in two swatches from a pair of socks submitted by eight park employees following three months of use (NC11 did not submit socks). Horizontal lines in each figure indicate the mean permethrin content in treated controls (dashed lines) and untreated controls (solid line)

**Fig. 2:**
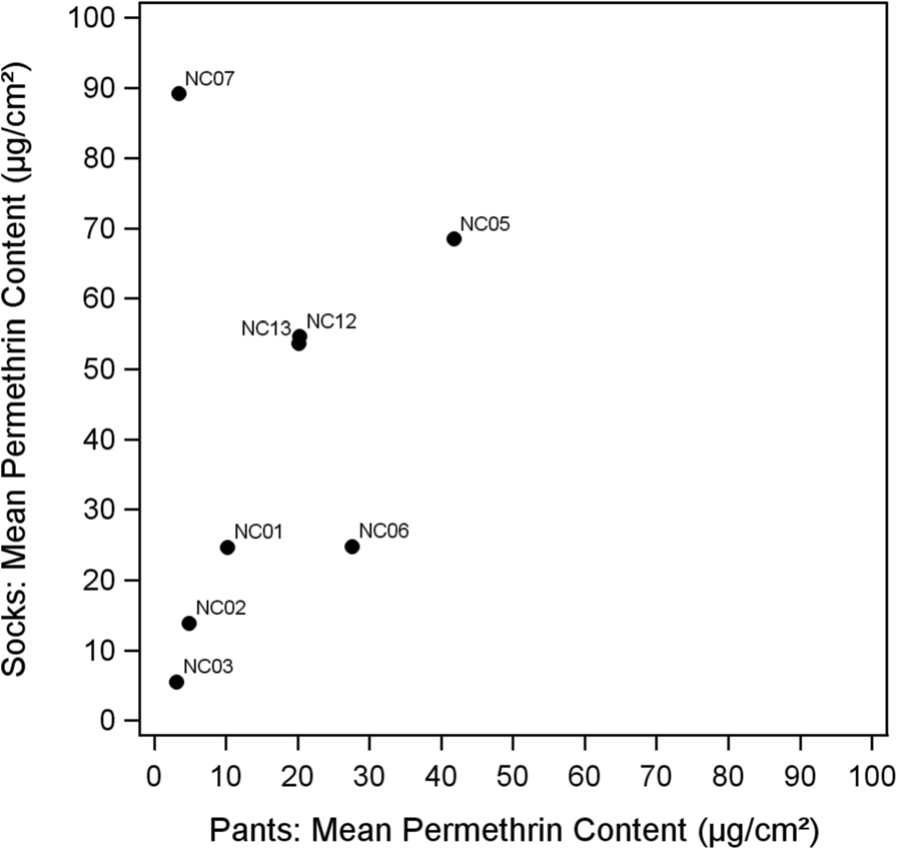
Mean permethrin content of pant and sock swatches after three months of wear by participant

Substantial variability in permethrin content was seen between participants in both pant and sock swatches (Fig. 1). Mean content ranged between 3.1–41.8 μg/cm^2^ in pants and between 5.5–89.1 μg/cm^2^ in socks. With the exception of participant NC07, mean permethrin contents between pants and socks tended to be correlated (*r* = 0.31, *n* = 8, *P* = 0.45; Fig. 2). Significant differences were observed in means of permethrin content between socks (*F*_(7,15)_ = 18.52, *P* = 0.0002) and pants (*F*_(8,26)_ = 163.40, *P* < 0.0001). Overall, socks had higher permethrin concentrations as compared with pants.

Swatches taken from the same article of clothing generally had similar permethrin contents within the three pant swatches and within the two sock swatches. However, within clothing variability was noted for several participants, including NC06 (pants) and NC01 (socks), NC07 (socks) and NC12 (socks).

### Tick mortality

**Fig. 3:**
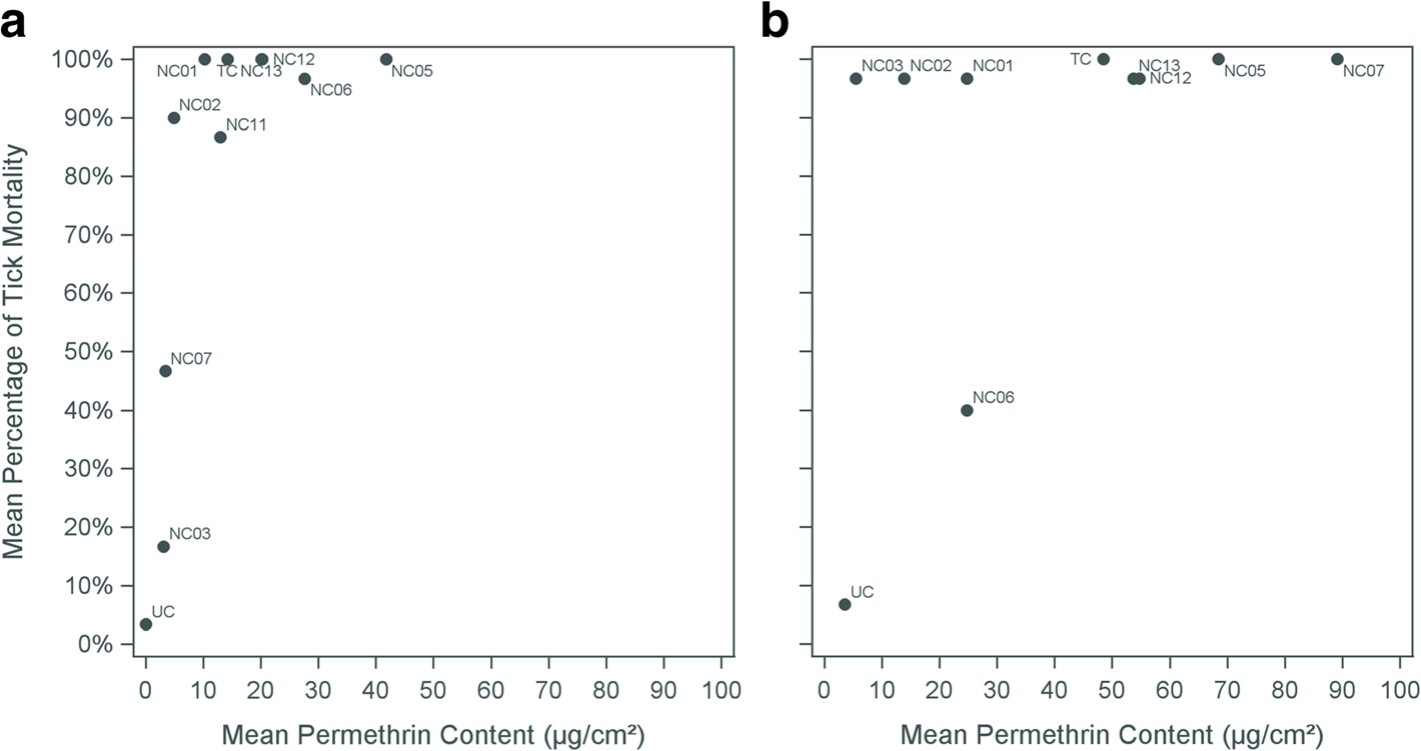
Mean permethrin content by mean visual tick mortality percentage after three months of wear. **a** Mean permethrin content by mean visual tick mortality percentage after three months of wear from three pant swatches tested. **b** Mean permethrin content by mean visual tick mortality percentage after three months of wear from two sock swatches tested. *Abbreviations*: TC, treated control; UC, untreated control

Overall, tick mortality was high for both clothing types (Fig. 3). From the visual tick inspection, 78% (7/9) of pant and 88% (7/8) of sock swatches tested had mean tick mortality percentages at or above 85%. Data points were few at lower permethrin content; however, there appeared to be a knockdown threshold at roughly 4 μg/cm^2^ permethrin. Below this level the median mean tick mortality percentage was 14% (*n* = 4), while above this level it was 97% (*n* = 17) among all swatches and controls.

### Mosquito knockdown and mortality

Overall, clothing swatches and treated control swatches did not effectively knock down or kill mosquitoes. Modest increased knockdown and/or mortality was seen against *A. albopictus* (permethrin susceptible) as compared with *Ae. aegypti* (permethrin resistant) (Table 2, Additional file 1: Figure S1 and Additional file 2: Figure S2) but these differences were not significant (*P* > 0.05). No knockdown or mortality was observed when mosquitoes were exposed to untreated swatches.

### Urine metabolites

Concentrations of 3-PBA, cis-DCCA, and trans-DCCA were measured in the urine of twelve participants (Table 1). 3-PBA was detected in 100% of the samples while cis-DCCA and trans-DCCA in 77% and 89%, respectively.

The average Permethrin Equivalent Daily Dose (PEDD, μg/kg/day) varied among participants. 3-PBA multi-month PEDD averages ranged between 0.32–7.12 (median 0.79), cis-DCCA between 0.12–4.64 (median 0.51), and trans-DCCA between 0.36–9.66 (median 1.08, Table 2). The PEDD estimated from the three metabolite concentrations were similar within participants. However, visual inspection of scatterplots did not show evidence of a relationship between PEDD and either mean sock or mean pant permethrin contents (Additional file 3: Figure S3).

For each individual, PEDD calculated from concentrations of all three metabolites were similar at one week and one-month (Table 1). After the three-month measurement, concentrations of all three metabolites had declined by more than two-fold. The calculated absorbed dosage of permethrin was less than approximately 4 μg of permethrin per kg of body weight.

## Discussion

In this pilot study conducted among outdoor workers, we found that LLPI clothing was both safe and remained highly effective at killing ticks following three months of use in real-world conditions. LLPI worn clothing did not effectively knockdown mosquitoes, however. This study provides additional evidence that the long-term use of LLPI clothing may be an appropriate and convenient supplementary measure for tick bite prevention among people with high risk of exposure to tick-borne diseases.

### Safety

From the urine biomarker results, the absorbed dosage of permethrin was < 4 μg/kg of body weight among LLPI wearers over the three-month period. According to Aylward et al*.* [12], the acceptable daily intake of permethrin in Europe is 50 μg/kg/d, while the USEPA [19] lists the chronic reference dose of permethrin as 250 μg/kg/d (based on an oral neurotoxicity study in rats). Thus, our study suggests that exposure for individuals wearing permethrin-impregnated clothing is more than an order of magnitude lower than the health protective goals established by regulatory agencies. It is also noteworthy that EPA (2009) lists a short-, intermediate- and long-term dermal applied dose (external exposure) No-Observed-Adverse-Effect-Level (NOAEL) of 500 mg/kg/day based on a 21-day dermal toxicity study in rats. In this study, a lowest observed adverse effect level was not established, indicating low dermal toxicity.

Baseline concentrations for two metabolites were within reference ranges reported among the US general population (National Health and Nutrition Examination Survey, NHANES [20]); NHANES does not include cis-DCCA. The range of 50th to 95th percentile in the US population for trans-DCCA in 20–59-year-olds is < LOD (0.6) to 5.88 μg/l. Similarly, the range of 50th to 95th percentile in the US population for 3-PBA in 20–59-year-olds is 0.39 to 6.95 μg/l [20].

### Permethrin retention

It has previously been shown that permethrin absorption increases with increased wear over short periods of time (days) [21]. Here, we measure a longer time frame and show that absorption remains constant between one week and one month of wear, and decreases by three months of wear.

Nevertheless, all swatches from worn clothing retained bioactive amounts of permethrin following three months of use. However, there was a large degree of inter-individual variation. Among both pant and sock swatches, we observed approximately a 15-fold difference in mean permethrin concentration between lowest and highest content swatches. The cause of these variations is unknown but could have been due to factors such as varying sunlight exposure or frequency of washing. However, given the relatively short duration of LLPI uniform use, we hypothesize that the observed variation of permethrin content may have been a result of variable permethrin application during the impregnation process.

In addition to the variation we observed between participant’s clothing, we also found that socks generally retained higher amounts of permethrin as compared with pants. This difference is likely explained by variation in permethrin adsorption during treatment due to fabric composition. Richards et al. [16] demonstrated that unwashed, LLPI treated 100% cotton fabrics had higher initial permethrin content than 50% cotton/50% polyester fabrics, with contents of 29.2 ± 2.9 μg/cm^2^ and 6.3 ± 1.0 μg/cm^2^, respectively.

Given the variability of permethrin content we observed between both clothing samples and clothing types, LLPI quality control measures should be considered to standardize the amount of permethrin-impregnated into clothing to maximize effectiveness and minimize unnecessary permethrin exposure.

### Tick and mosquito mortality

The observed variability in permethrin content did not significantly impact tick mortality, as clothing swatches remained highly effective at killing ticks following three months of use. In our laboratory analyses, a median of 97% mean mortality was seen among all clothing samples tested. This laboratory result is consistent with previous laboratory and field studies which found LLPI clothing to be highly effective at preventing tick bites [11, 22–25].

Swatches of treated fabric were ineffective against mosquitoes in laboratory assays for knockdown and/or mortality. However, a recent study suggests that the protective efficacy of treated clothing is due to repellency rather than lethality [26]. Evidence for the effectiveness of LLPI clothing in preventing mosquito bites was also demonstrated by a study showing lower antibody titers to mosquito salivary proteins among field workers wearing LLPI clothing [27]. Thus, while LLPI clothing worn for three months is not lethal to mosquitoes, it may remain effective against mosquitoes through repellency. Future studies should consider this distinction.

### Strengths and limitations

Our study has several strengths. First, we monitored the bioabsorption of permethrin in LLPI users at four time points over a period of three months. Previous studies which have demonstrated the safety of LLPI clothing evaluated permethrin metabolites at a single time point or over a short-term period. Our study extends current safety knowledge by providing longer-term profiles of permethrin bioabsorption and metabolite excretion in urine. Secondly, our study comprehensively evaluated LLPI clothing by combining safety data and with tick mortality effectiveness among outdoor workers using LLPI clothing under normal wear and laundering. To date, studies of LLPI effectiveness have primarily evaluated tick bite prevention as measured by self-report in field studies.

This study is subject to at least four limitations. First, this was a small pilot study with only 13 participants, therefore findings should be interpreted cautiously. Secondly, although the duration of follow-up was longer than previous studies, we followed participants for only three months. As LLPI clothing is believed to retain bioactive amounts of permethrin for up to 70 washes (the expected lifetime of the garment), we expect loss of knockdown effectiveness over longer periods of time than that studied. Faulde et al. [28] demonstrated > 98% permethrin loss over 100 launderings. Results from our study, therefore, cannot provide information regarding the time at which LLPI clothing becomes ineffective against ticks under normal use. Thirdly, we were unable to measure permethrin content of clothing following impregnation as clothing was sent directly to study participants after impregnation. Although we believe that the impregnation process may have contributed to the variation in the retained content following three months, we are unable to directly demonstrate this using these data. Finally, the cis- and trans-DCCA are specific, cyfluthrin, cypermethrin or permethrin, and 3-PBA is a metabolite common to eight different pyrethroids. Despite the potential for misclassification of the source of the metabolites, the fact that all three metabolites rose and fell in concert following wear of LPPI clothing strongly implicates permethrin as the source of the increased metabolite concentrations.

## Conclusions

In conclusion, effective levels of permethrin are retained by impregnated clothing for up to three months while exposure to those wearing the clothing remains well below recommended levels. The consistency of fabric treatment may vary. Further studies on efficacy and safety over longer periods of time are needed.

## Additional files

Additional file 1: **Figure S1.** Mean permethrin content by mean *Ae*. *albopictus* knockdown (2 h) and mortality (24 h). **a** Mean permethrin content in pants by mean *Ae. albopictus* knockdown at 2 h. **b** Mean permethrin content in socks by mean *Ae. albopictus* knockdown 2 h. **c** Mean permethrin content in pants by mean *Ae. albopictus* mortality at 24 h. **d** Mean permethrin content in socks by mean *Ae. albopictus* mortality at 24 h. (TIF 2345 kb)

Additional file 2: **Figure S2.** Mean permethrin content by mean *Ae. aegypti* knockdown (2 h) and mortality (24 h). **a** Mean permethrin content in pants by mean *Ae. aegypti* knockdown at 2 h. **b** Mean permethrin content in socks by mean *Ae. aegypti* knockdown 2 h. **c** Mean permethrin content in pants by mean *Ae. aegypti* mortality at 24 h. **d** Mean permethrin content in socks by mean *Ae. aegypti* mortality at 24 h. (TIF 2298 kb)

Additional file 3: **Figure S3.** Mean permethrin content by multi-month average of three urine metabolites. **a** Mean permethrin content in pants by multi-month 3-PBA average. **b** Mean permethrin content in socks by multi-month 3-PBA average. **c** Mean permethrin content in pants by multi-month trans-DCCA average. **d** Mean permethrin content in socks by multi-month trans-DCCA average. **e** Mean permethrin content in pants by multi-month cis-DCCA average. **f** Mean permethrin content in socks by multi-month cis-DCCA average. (TIF 2819 kb)
