# Supplementary figures and images for: Bioabsorption and effectiveness of long-lasting permethrin-treated uniforms over three months among North Carolina outdoor workers
Source: Parasit Vectors. 2019 Jan 23;12:52. doi: 10.1186/s13071-019-3314-1 (PMC6343280; doi:10.1186/s13071-019-3314-1)

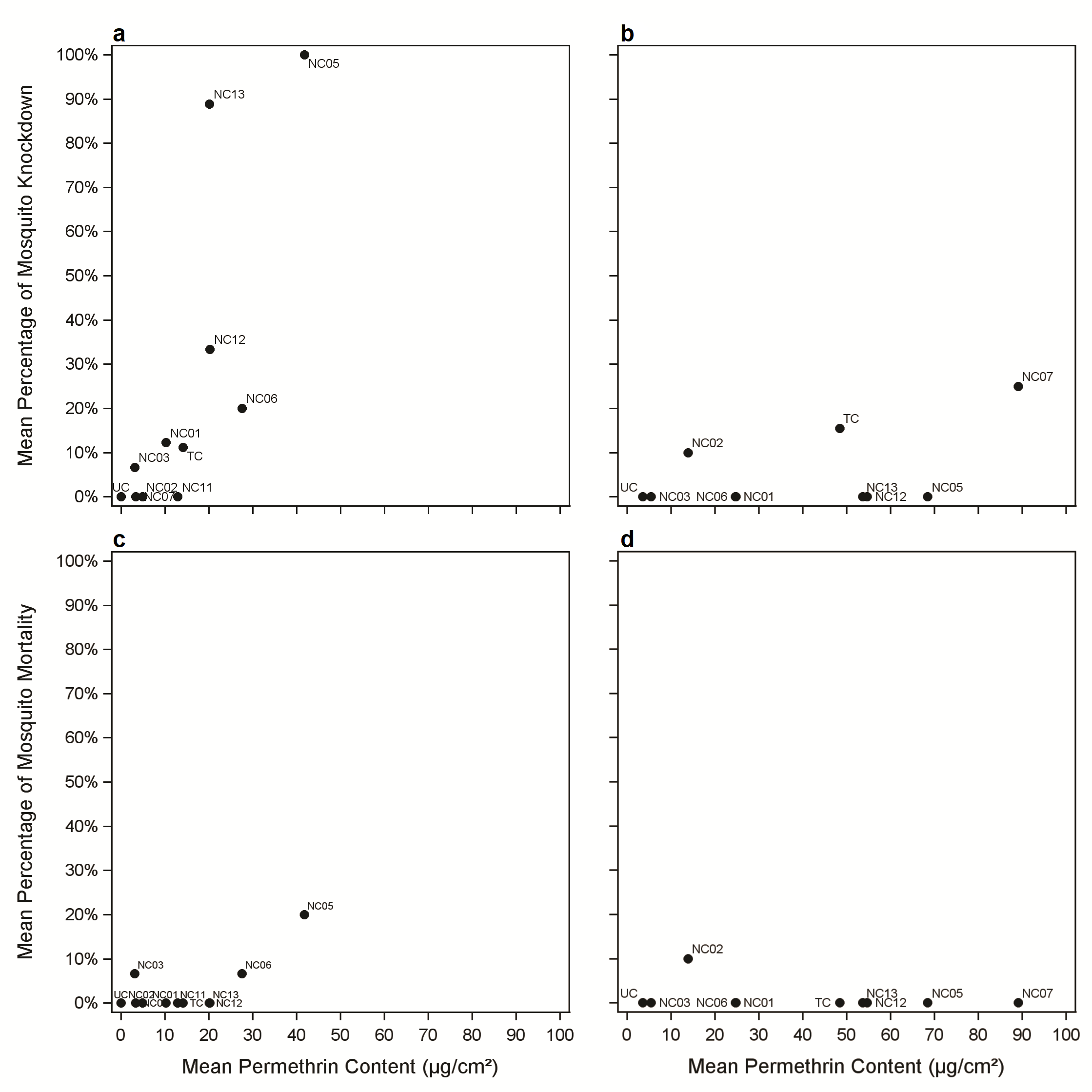

Supplement: Supplementary file 1 — Figure S1. Mean permethrin content by mean Ae. albopictus knockdown (2 h) and mortality (24 h). a Mean permethrin content in pants by mean Ae. albopictus knockdown at 2 h. b Mean permethrin content in socks by mean Ae. albopictus knockdown 2 h. c Mean permethrin content in pants by mean Ae. albopictus mortality at 24 h. d Mean permethrin content in socks by mean Ae. albopictus mortality at 24 h. (TIF 2345 kb) [file 13071_2019_3314_MOESM1_ESM.tif]

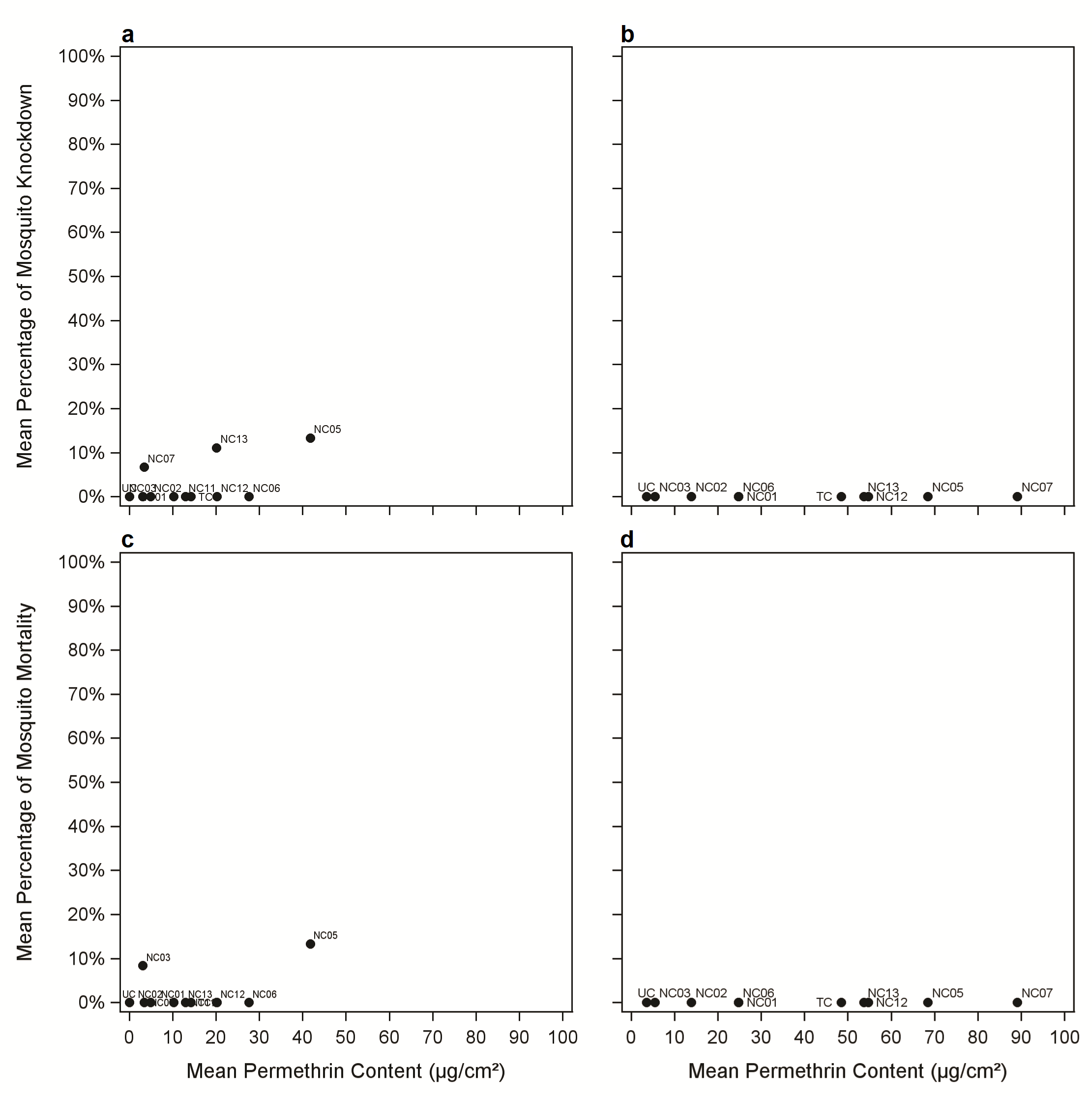

Supplement: Supplementary file 2 — Figure S2. Mean permethrin content by mean Ae. aegypti knockdown (2 h) and mortality (24 h). a Mean permethrin content in pants by mean Ae. aegypti knockdown at 2 h. b Mean permethrin content in socks by mean Ae. aegypti knockdown 2 h. c Mean permethrin content in pants by mean Ae. aegypti mortality at 24 h. d Mean permethrin content in socks by mean Ae. aegypti mortality at 24 h. (TIF 2298 kb) [file 13071_2019_3314_MOESM2_ESM.tif]

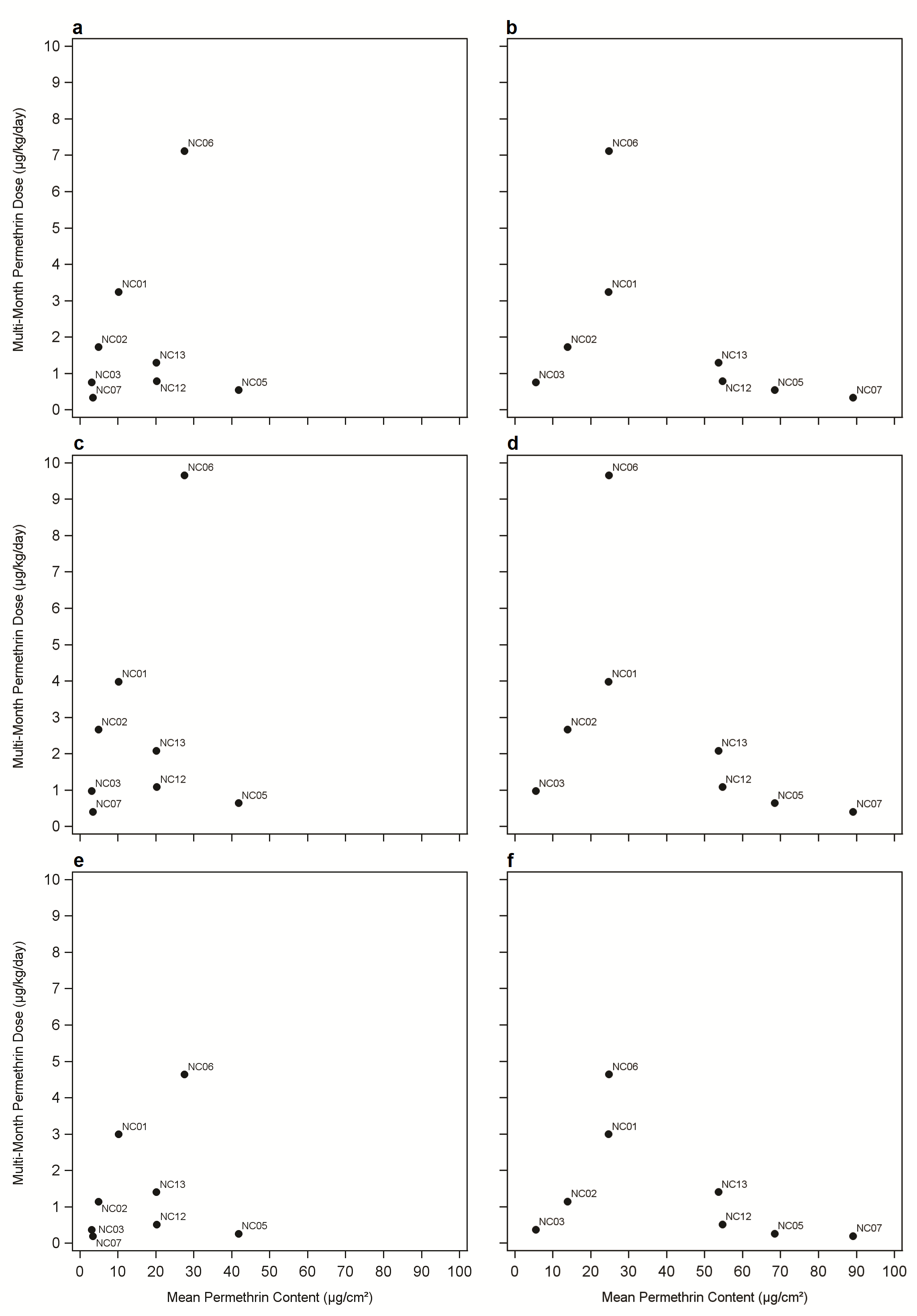

Supplement: Supplementary file 3 — Figure S3. Mean permethrin content by multi-month average of three urine metabolites. a Mean permethrin content in pants by multi-month 3-PBA average. b Mean permethrin content in socks by multi-month 3-PBA average. c Mean permethrin content in pants by multi-month trans-DCCA average. d Mean permethrin content in socks by multi-month trans-DCCA average. e Mean permethrin content in pants by multi-month cis-DCCA average. f Mean permethrin content in socks by multi-month cis-DCCA average. (TIF 2819 kb) [file 13071_2019_3314_MOESM3_ESM.tif]
